# Axiomatic Design of a Framework for the Comprehensive Optimization of Patient Flows in Hospitals

**DOI:** 10.1155/2017/2309265

**Published:** 2017-06-19

**Authors:** Gabriele Arcidiacono, Dominik T. Matt, Erwin Rauch

**Affiliations:** ^1^Department of Innovation and Information Engineering, Guglielmo Marconi University, Via Plinio 44, 00193 Rome, Italy; ^2^Faculty of Science and Technology, Free University of Bozen-Bolzano, Piazza Università 5, 39100 Bolzano, Italy; ^3^Fraunhofer Italia Research s.c.a.r.l., Innovation Engineering Center (IEC), Via Macello 57, 39100 Bolzano, Italy

## Abstract

Lean Management and Six Sigma are nowadays applied not only to the manufacturing industry but also to service industry and public administration. The manifold variables affecting the Health Care system minimize the effect of a narrow Lean intervention. Therefore, this paper aims to discuss a comprehensive, system-based approach to achieve a factual holistic optimization of patient flows. This paper debates the efficacy of Lean principles applied to the optimization of patient flows and related activities, structures, and resources, developing a theoretical framework based on the principles of the Axiomatic Design. The demand for patient-oriented and efficient health services leads to use these methodologies to improve hospital processes. In the framework, patients with similar characteristics are clustered in families to achieve homogeneous flows through the value stream. An optimization checklist is outlined as the result of the mapping between Functional Requirements and Design Parameters, with the right sequence of the steps to optimize the patient flow according to the principles of Axiomatic Design. The Axiomatic Design-based top-down implementation of Health Care evidence, according to Lean principles, results in a holistic optimization of hospital patient flows, by reducing the complexity of the system.

## 1. Introduction

Health Care organizations today are facing a new and unique set of challenges bringing them under pressure to obtain efficiency improvements and cost reduction. Expenditures have grown steadily, and budgets are increasingly being shortened while requested services and expected quality continue to increase. To overcome these challenges in a sustainable way, Health Care organizations need to improve their services in terms of costs, response times, and service quality as well as resource utilization. Similar challenges occur also in other industries like manufacturing, where Lean Management approaches showed promising results and potential in the reduction of inefficiencies and in increasing value for the customer.

The great success of Lean Management [1] and Six Sigma [2] in industry has led to the fact that its application is no longer limited to the manufacturing industry, but is extended to service industry and public administration. Given the growing demand for patient-oriented and efficient health services, Lean Management and Six Sigma methods are now increasingly used also in hospitals [3–6].

The bibliometric investigation of Walshe [3] shows emerging trends for quality improvement in hospitals based on citations over a time period of 20 years. The study illustrates that there are three major quality improvement methods treated in literature: (a) Lean, (b) Six Sigma, and (c) Patient Safety. In the study, these three methods are two to three times as important as other methods like accreditation, process redesign, or clinical governance. Arcidiacono et al. [4] describe in their Lean Six Sigma project study the key factors to improve operating room utilization by the use of Lean methods and Six Sigma methods. Brandao de Souza [5] investigates new trends and approaches in Lean Health Care. According to the results of his study, there exists an agreement about the potential of Lean Health Care, but it remains a challenge for academics and practitioners to evaluate Lean Health Care under a more comprehensive and practical perspective. Coronado and Anthony [6] analyze the critical success factors for the effective implementation of Six Sigma projects in organizations. In addition to many other prerequisites like training, communication, or cultural change, also a comprehensive approach and introduction are important factors for succesful projects. According to Radnor [7], organisational readiness is important for a sustainable implementation of Lean in Health Care, including the understanding of the process/system view, customer view, data, and engaging the staff to ensure that Lean is not just about making poor processes more efficient by focusing on the tools.

However, in many cases treated by literature references, lean principles were applied in single departments or to solve specific problems. A comprehensive study and investigation of how to optimize patient flows in a hospital is still missing. The motivation of this paper is to close this gap in research and to give an example how an Axiomatic Design-based approach helps to reduce complexity in the Health Care environment. The paper aims to provide an original and innovative set of design guidelines in order to support practitioners and researchers in the design of effective and efficient patient flows in hospitals.

From a methodological point of view, this paper aims at introducing a comprehensive, system-based approach as an integrated and practically proven model and methods package for the implementation of a Lean Health Care delivery operation. The methodology discussed throughout the paper is grounded both in a review of the literature available on the subject and in evidence taken from the experience of the authors in Lean and, specifically, with analyses based on the application of Axiomatic Design theory.

## 2. Methods: Axiomatic Design and Lean Management in Health Care

Lean Management applied to Health Care has raised the question of what are true value activities from the patient's perspective, that is, which activities contribute to promote and enhance the patients' well-being and/or their recovery. Based on this approach, all activities distributed along the entire patient flow—from admission to discharge—that do not offer a value-added contribution are questioned [7].

In this context, it must be noted that the introduction of Lean Management through the selective application of specific tools in individual processes is not truly effective [8], as a structured approach is needed in order to capture the complexity of the entire system [9] and to make it manageable [10, 11]. First research approaches on Lean thinking applied to Health Care focused on how the concept of holistic production systems was transferred from industry to hospitals [12–14]. To date, however, literature does not report any effective demonstration of such holistic optimization of the hospital system according to Lean Six Sigma [15, 16].

Therefore, this paper develops a theoretic framework for the target-oriented (re)design of patient flows in a North Italian city hospital. The study is based on the principles of the Axiomatic Design theory [17], which is investigated here through its practical relevance in Health Care-based evidences.

Axiomatic Design has proven its success in reducing or managing the complexity [18] of many systems. Axiomatic Design is applicable to various kinds of systems [19, 20]. This has led researchers to extend the use of this approach also to Health Care system optimization [10, 21, 22]. Axiomatic Design is most successfully applied when it comes to the design of a complex system that is too difficult to understand in an intuitive way. By creating a design matrix of the system top-to-bottom, it helps to define the system objectives and to find suitable design solutions in a structured manner [17].

Suh defines system complexity as a measure of uncertainty in achieving a set of design targets that a system must fulfil within a given range of tolerance [23].

According to this definition, the complexity of a system is determined by two factors [24]: by a time-independent poor design, which results in a systemic low efficiency (system design), and by a time-dependent decrease in system performance due to environmental changes or system deterioration (system dynamics).

System complexity is low if the design targets can be met within a specified tolerance. Thus, to reduce the time-independent system's complexity, the first step is to clearly define its goals or requirements and the related tolerances. The definition and the subsequent decomposition of the system targets usually starts from the system's stakeholder perspective. Regarding a Health Care delivery system, stakeholders are primarily patients and medical and nursing staff, but also an economic perspective and certain legal requirements, rules and regulations, or other important surrounding conditions have to be considered. For each level of the system objectives, appropriate solutions to the mutually exclusive and collectively exhaustive fulfilment of these design objectives have to be found. This is what Axiomatic Design as an approach for system design performs in a very efficient and systematic way.

**Figure 1:**
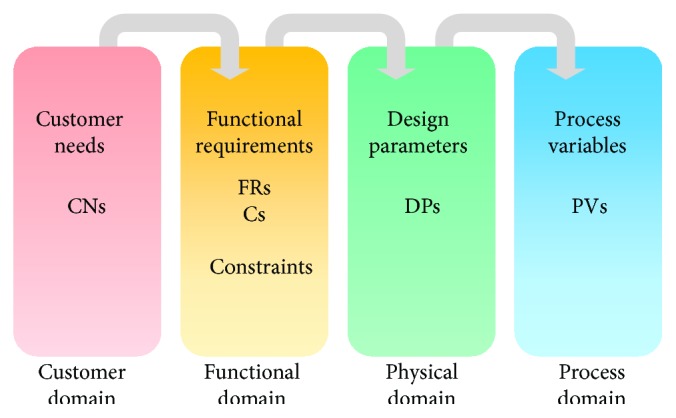
The four domains of the Axiomatic Design world.

According to Axiomatic Design [17], we differentiate between four different design domains (Figure 1):

- Customer Domain with Customer Needs (CNs)
- Functional Domain with Functional Requirements (FRs) and Constraints (Cs)
- Physical Domain with Design Parameters (DPs)
- Process Domain with Process Variables (PVs).

**Figure 2:**
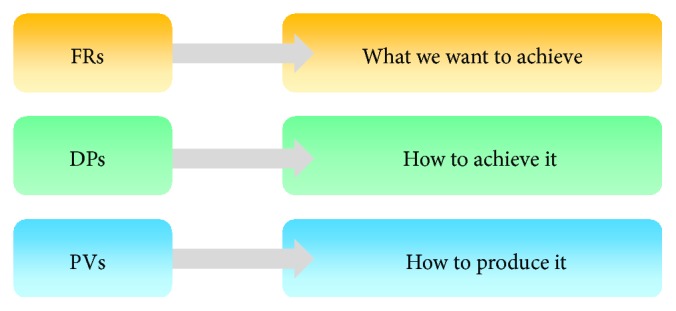
Meaning of the different variables related to the domains.

The Customer Domain is characterized by CNs that can be explained as a simple wish list of the customer regarding to his expectations regarding the system. They have to be transformed then into a minimum set of independent requirements that completely characterize the functional needs of the product, the FRs. In the Functional Domain, the CNs are specified in terms of FRs and Cs. Constraints or Cs are defined as bounds on acceptable solutions (e.g., a maximum budget or a minimal and maximal value for a parameter). In order to satisfy the specified FRs, we have to identify and choose DPs in the Physical Domain. Finally, to realize the system specified in terms of DPs, a process, characterized by PVs in the Process Domain, has to be developed (Figure 2).

The Customer Domain deals with the Customer Needs (CNs) or Attributes (or of other stakeholders of the system) regarding product, process, system, or other design objects. In the Functional Domain, CNs are translated into Functional Requirements (FRs) which represent the actual targets of the design at a certain point in time. The Design Parameters (DPs) in the Physical Domain serve to satisfy the FRs. For the realization of the design solution specified by the Design Parameters, the Process Variables (PVs) finally are defined in the Process Domain [17]; however, in most cases, system design stops after the FR-DP mapping.

In nearly all design tasks, the problem must be further decomposed. The FR-DP hierarchy is developed by a top-down decomposition and mapping process between two domains, called also “zig-zagging” in Axiomatic Design [17]. This means, that after having assigned the DP to the FR at the top level, the next FR level has to be decomposed based on the chosen design solution at the previous level. Then FR-DP mapping continuous assigning the DPs to the chosen FRs, and so on.

Two axioms guide the system designer within mapping between the domains. They help to evaluate and select design options in order to produce a robust design. The two axioms in Axiomatic Design defined by Suh [17] are as follows:

- Axiom 1: The Independence Axiom. It postulates to maintain the independence of the FRs. With two or more FRs, a design solution must be found that allows each one of the FRs to be satisfied without affecting the other FRs.
- Axiom 2: The Information Axiom. It claims the minimization of the design's information content. The design with the least amount of information is the most suitable to fulfil the FRs.

The FRs and DPs are represented mathematically as a vector. The Design Matrix [*A*] shows the relationship between FRs and DPs [17]:

(1) $$\begin{array}{c} \left\{FRs\right\}=\left[A\right]\left\{DPs\right\}. \end{array}$$

In the expanded form,

(2) $$\begin{array}{c} \left[\begin{array}{c} {FR}_{1}\\ {FR}_{2}\\ \vdots \\ {FR}_{m} \end{array}\right]=\left[\begin{array}{cccc} A_{11} & A_{12} & \cdots & A_{1n}\\ A_{21} & A_{22} & \cdots & A_{2n}\\ \vdots & \vdots & \ddots & \vdots \\ A_{m1} & A_{m2} & \cdots & A_{mn} \end{array}\right]\cdot \left[\begin{array}{c} {DP}_{1}\\ {DP}_{2}\\ \vdots \\ {DP}_{n} \end{array}\right], \end{array}$$

where the element *A*_*ij*_ of the matrix represents the relationship between FR_*i*_ and DP_*j*_.

The Independence Axiom requires the Design Matrix to be diagonal or triangular. In a diagonal Design Matrix, exactly one DP can fulfil each of the FRs independently. It is called an uncoupled system design. In a triangular matrix, the independence of FRs can be fulfilled only if the DPs are determined in a proper sequence. Such a design is called a decoupled design. Any other form of the Design Matrix results in a coupled system design [17].

However, even in an uncoupled or decoupled design, the once selected DPs may—over time—move outside the originally defined FR design range. Similarly, also the system targets may change over time. This so-called time-dependent system complexity has its origins in the unpredictability of future events that might change the current system. According to Suh [23], the time-dependent complexity can be only controlled by introducing a functional periodicity [24]. In practical terms, this means to regularly “reset” the system, for example by the implementation of a Continuous Improvement Process or Kaizen [25].

## 3. Results

### 3.1. Patient Flows in a Hospital

**Figure 3:**
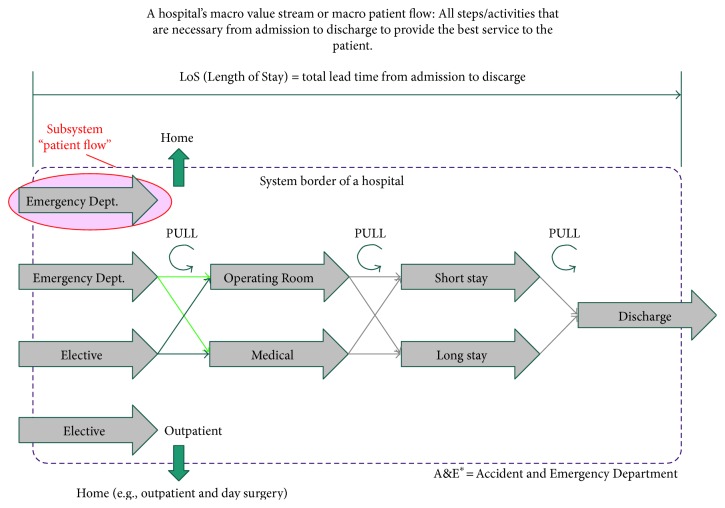
Patient flows in the hospital system [27].

Health Care delivery systems in general and more specifically hospitals are—like organizational systems in general—collections of persons, devices, and processes organized to accomplish all the necessary operations [26] to ensure the recovery or the improvement of the patient's health condition. According to system theory, every system may be described as an aggregation of subsystems [17]. Consequently, a Health Care delivery system can be considered an assemblage of single (process) units along the system's value stream (Figure 3).

In the context of Health Care, we replace the term “value stream” with “patient flow”. As shown in Figure 3, the overall patient flow—or “macro patient flow”—goes similarly to an industrial value stream that ranges from “ramp to ramp,” from the entrance to the system hospital to the exit, that is, from admission to discharge.

The route that the single patient takes depends on several factors, for example, disease pattern, bed capacities, and hospital specialization. However, any patient route may be mapped as an assemblage of single patient flows, such as “Emergency Dept. + Operating Room + Short Stay + Discharge” or others. Accordingly, the overall “macro” Length of Stay (LoS) as the sum of the single patient flows lead time changes depending on the single patient's route. The single patient flows like “Emergency Dept.” are subject to specific characteristics, performances, and figures. For instance, an emergency department usually has much more patients to manage than the subsequent patient flow “operating room.” Due to these differences and a given high variety of patient cases, it is not possible to define “one” whole macro patient flow. The single patient flows have to be decoupled and synchronized, for example, by buffers that are managed according to the Pull-principle [27], in order to achieve the shortest possible macro LoS.

A patient flow typically starts and ends at the system borders of a single department, such as emergency department, a medical ward, or discharge. Due to the diversity of cases, it might be useful to cluster patients with similar characteristics into so-called patient families in order to achieve homogeneous patient flows [22]. A helpful tool for the design of patient flows in a Health Care delivery system is value stream mapping, a key instrument of the Lean toolbox, adapted to the specific characteristics of patient flows [22, 28].

The following sections will focus on the subsystem “patient flow.”

### 3.2. Mapping and Decomposition Using Axiomatic Design in Health Care

Probably the most critical phase in Axiomatic Design is the determination of the first level of FRs. It requires a very accurate study of Customer Needs with respect to the system design. In the case of a hospital, these needs are addressed primarily by patients, but also by staff and by a large set of economic objectives, legal requirements, administrative regulations, and surrounding conditions that the system has to fulfil.

Baker et al. [28] identified a basic set of 5 superior objectives (CNs) for a hospital:

- CN-1: Enhance the emergency access performance (i.e., reduce the lead time in A&E Accident and Emergency Department).
- CN-2: Improve access to scheduled services (i.e., reduce the patients' waiting time from referral to treatment).
- CN-3: Reduce cost (i.e., close beds and, consequently, reduce staff).
- CN-4: Reduce medical LoS.
- CN-5: Reduce rates of Hospital Acquired Infections (HAI).

The translation of the CNs in FRs is very important and difficult at the same time, because the quality of the further design is built upon the integrity and accuracy of the selected CNs. The Independence Axiom requires the independence of the chosen FRs. This must be considered when translating the main superior CNs into the highest-level FR(s).

Baker et al. [28] state that the five CNs are related, as they all refer to the LoS: the longer the LoS, the more patients are in the system (hospital), the more beds are occupied, the more cost is generated, the more stress, and the more errors occur which again leads to an increase in LoS. This is true for CN-1, CN-2, and CN-5. However, costs are only partly affected by LoS reduction. On the contrary, the reduction of lead times may at certain points even increase cost, especially when it comes to eliminate bottlenecks. Another example are necessary one-time expenditures that might improve quality without being related to LoS.

First of all, we begin the decomposition and mapping process in Axiomatic Design starting from FR-0 to DP-0 level:

- FR-0: Optimize efficiency of patient flows in hospitals.
- DP-0: Realize a short and cost-efficient stay of patients.

DP-0 is on a very abstract level and therefore has to be further decomposed and splitted in the following first-level FRs:

- FR-1: Realize the required patient throughput within the shortest possible lead time.
- FR-2: Realize the lowest possible costs per patient.

The selected DPs are as follows:

- DP-1: The patient flow provides the required output according to Lean Six Sigma principles.
- DP-2: The design of the patient flow provides the lowest possible total cost per patient.

The Design Matrix is uncoupled:

(3) $$\begin{array}{c} \left\{\begin{array}{c} FR1\\ FR2 \end{array}\right\}=\left[\begin{array}{cc} X & 0\\ 0 & X \end{array}\right]\left\{\begin{array}{c} DP1\\ DP2 \end{array}\right\}, \end{array}$$

with *X* as a nonzero element and 0 as a zero element.

The selected design solution cannot be completed at the highest level. Therefore, the FRs have to be decomposed further by zigzagging between the two domains.

Starting from DP-1, the next FR level is determined as follows:

- FR-11: Produce to required output rate.
- FR-12: Establish a continuous flow.
- FR-13: React quickly and cost efficiently to unplanned problems.
- FR-14: Avoid periods of poor workload.
- FR-15: Achieve operational flexibility.

We assign the following DPs (DP-11, DP-12, DP-13, DP-14, and DP-15) to the single FR (FR-11, FR-12, FR-13, FR-14, and FR-15):

- DP-11: Determine the takt time
- DP-12: Balance station cycle times to the takt time
- DP-13: Visual control and fast intervention concept
- DP-14: Workload optimized scheduling
- DP-15: Reduction of “setup times.”

The design is decoupled:

(4) $$\begin{array}{c} \left\{\begin{array}{l} FR11\\ FR12\\ FR13\\ FR14\\ FR15 \end{array}\right\}=\left[\begin{array}{lllll} X & 0 & 0 & 0 & 0\\ X & X & 0 & 0 & 0\\ 0 & 0 & X & 0 & 0\\ 0 & 0 & 0 & X & 0\\ 0 & 0 & 0 & 0 & X \end{array}\right]\left\{\begin{array}{l} DP11\\ DP12\\ DP13\\ DP14\\ DP15 \end{array}\right\}. \end{array}$$

As next, FR-2/DP-2 will be decomposed further:

- FR-21: Achieve lowest error rates.
- FR-22: Minimize labor costs.
- FR-23: Minimize one-time expenditures.

The Design Parameters for FR-21, FR-22, and FR-23 are as follows:

- DP-21: Process quality and stability optimized according to Lean Six Sigma principles
- DP-22: Effective use of workforce
- DP-23: Selection of modular system components wherever possible.

The design is again decoupled:

(5) $$\begin{array}{c} \left\{\begin{array}{c} FR21\\ FR22\\ FR23 \end{array}\right\}=\left[\begin{array}{lll} X & 0 & 0\\ 0 & X & 0\\ X & 0 & X \end{array}\right]\left\{\begin{array}{c} DP21\\ DP22\\ DP23 \end{array}\right\}. \end{array}$$

Decomposition of FR-21 refers mainly to the probability that an error occurs and that it is passed to the next station:

- FR-211: Provide error-free input.
- FR-212: Prevent making errors throughout the process.
- FR-213: Do not advance errors to the next patient flow(s).

The selected DPs are as follows:

- DP-211: Initial quality check prior to process start
- DP-212: Use of standards and devices to prevent errors
- DP-213: Use of in-process and successive checks to identify and eliminate errors.

The matrix shows an uncoupled design:

(6) $$\begin{array}{c} \left\{\begin{array}{c} FR211\\ FR212\\ FR213 \end{array}\right\}=\left[\begin{array}{lll} X & 0 & 0\\ 0 & X & 0\\ 0 & 0 & X \end{array}\right]\left\{\begin{array}{c} DP211\\ DP212\\ DP213 \end{array}\right\}. \end{array}$$

The next decomposition of FR-22 has the purpose to reduce labor costs through the improvement of workplace and work time organization, ergonomics, and motivation:

- FR-221: Eliminate or reduce nonvalue-adding activities.
- FR-222: Enable (medical and nursing) staff to operate more than one station.
- FR-223: Plan resources to work efficiently with different patient volumes.

Effective DPs to implement FR-221, FR-222, and FR-223 may be selected as

- DP-221: Short distances to avoid/eliminate wasted handling or movement
- DP-222: Multifunctional staff (qualification and job rotation)
- DP-223: Work time flexibility in combination with a direct and measurable relation between output and work hours of resources.

Also, this matrix shows an uncoupled design:

(7) $$\begin{array}{c} \left\{\begin{array}{c} FR221\\ FR222\\ FR223 \end{array}\right\}=\left[\begin{array}{lll} X & 0 & 0\\ 0 & X & 0\\ 0 & 0 & X \end{array}\right]\left\{\begin{array}{c} DP221\\ DP222\\ DP223 \end{array}\right\}. \end{array}$$

Summarizing, it can be stated that the chosen FR-DP hierarchy represents a good design as it fulfils the Independence Axiom. However, the overall matrix is not completely uncoupled but triangular which implies that the optimization procedure has to strictly follow the sequence along the FR-DP leaf level of hierarchy to reduce complexity in the system design.

### 3.3. The Optimization Checklist for Patient Flows in Hospitals

**Figure 4:**
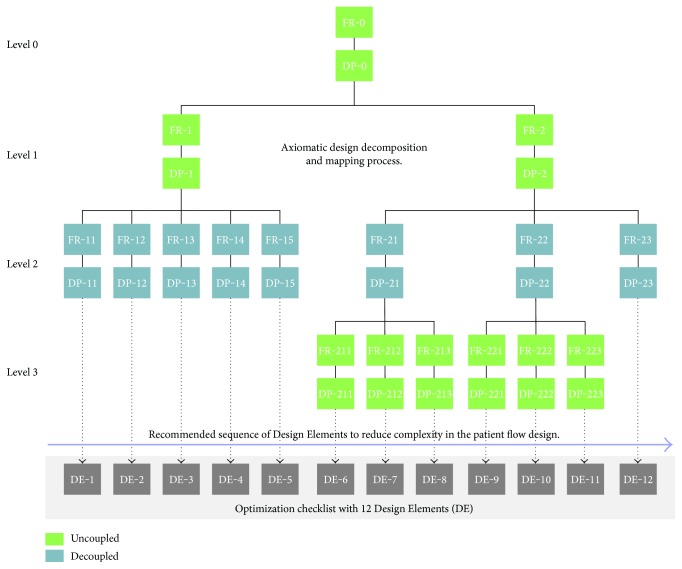
Axiomatic Design-based decomposition and mapping process to identify a list of 12 Design Elements for optimized patient flows in hospitals.

As a result of the FR-DP mapping conducted so far, an optimization checklist with a sum of 12 Design Elements (DE) for the design of optimized and efficient patient flows has been developed (Figure 4).

This checklist outlines the exact sequence of each single DE required to achieve an optimized patient flow:

- DE-1: Determine the required output rate (takt time) as an indication for determining the pace for every station in the process.
- DE-2: Create a continuous flow balancing the station cycle times to the pace of incoming patients (takt time).
- DE-3: Implement a visual control and a fast intervention strategy to solve unplanned problems.
- DE-4: Introduce a workload optimized scheduling.
- DE-5: Reduce “setup times” (preparation of rooms, instruments, etc.).
- DE-6: Ensure an uninterrupted supply of defect-free input material by an initial quality check prior to process start.
- DE-7: Use suitable standards and devices to prevent errors throughout the single processes in the patient flow.
- DE-8: Do not advance errors to the next station or to the subsequent patient flow.
- DE-9: Eliminate or reduce nonvalue-adding activities.
- DE-10: Enable (medical and nursing) staff to operate more than one station.
- DE-11: Ensure flexibility to accommodate capacity increments at lowest cost.
- DE-12: Minimize one-time expenditures by investing preferably in modular system components.

## 4. Discussion

As announced in the introduction, this paper aims to develop and provide a list of design guidelines for optimized patient flows in hospital. To guarantee a scientific approach and to reduce complexity, the research team decided to use an Axiomatic Design-based approach as research methodology.

Such checklist acts as fundamental output to answer the research question on which this research was based, because it allows a systematic organization of patient flow(s) and related activities. Most importantly, it is a flexible checklist, of which only certain steps might be selected according to the specific features of the process system(s) to which it is applied. Moreover, it is easily understandable and applicable by hospital operators at all levels of the hospital system, which makes it a useful tool to manage processes in view of a holistic optimization of the whole system itself. According to the Axiomatic Design theory, the decoupled design matrix proposes an ideal sequence for implementing the lowest level DPs (called Design Elements) reading Figure 4 from the left to the right.

The determination of the output rate (DE-1) defines in a first step the pace for every station in the whole Health Care process. As in every other system with a certain throughput rate, this is of highest importance to align the single-process steps along the LoS to the so-called “patient takt time.” This alignment and balancing is defined in DE-2 and needs a detailed analysis of the single stations and their cycle times. To solve unplanned problems, a fast intervention is needed (DE-3). This can be achieved by implementing monitoring systems and a visual control of deviations for medical and nursing staff. According to DE-2, the workload of every station needs to be optimized and scheduled through a planning and scheduling system (DE-4). To implement DE-2 and DE-4, hospitals need advanced planning and monitoring IT systems connected to the general ERP (Enterprise Resource System) of the hospital. Although to the past and ongoing developments in this area, further research is needed to optimize existing systems and to facilitate the digital transformation in Health Care. DE-5 describes the need for reduction of setup times for operating rooms or important bottleneck stations. In the Lean Theory, many existing and proven concepts for reduction of changeover times (e.g., Single Minute Exchange of Die—SMED) can be transferred and applied. Also for DE-6 to DE-9, many instruments and concepts for supplier development, quality improvement, and reduction of not value-adding activities can be transferred from Lean and Six Sigma theory to Health Care. DE-10 is looking for multifunctional staff to operate more than one station. This DE requires changes in internal training as well as educational programmes foreseeing a certain flexibility of medical and nursing staff. The same occurs for DE-11 looking for work time flexibility; this requires probably organizational changes and discussions with employee representatives. An investment in flexible, scalable, and modular machines or devices (DE-12) to reduce one-time expenditures is a highly discussed issue in production science. In this case, many concepts regarding to agile and sustainable systems exist.

However, the comprehensive and holistic optimization of patient flows in a hospital is a complex initiative that requires first the commitment of hospital governance as well as from medical and nursing staff. Therefore, the authors suggest, based on practical Lean and Six Sigma projects and experiences in different hospital organizations, to start any initiative with an orientation phase addressed to inform all involved stakeholders about the objectives and to give an overview about Lean and Six Sigma approaches and best practices. This reduces scepticism and helps to raise awareness for the need of changes in culture as well as operational and organizational processes.

## 5. Conclusion

The theoretical and methodological framework discussed so far has allowed to reduce and manage effectively the complexity of the system under observation, through the top-down implementation of Axiomatic Design on Health Care-based evidences. Namely, the application of the Axiomatic Design for the comprehensive optimization of patient flows according to Lean Six Sigma principles allowed a factual holistic optimization of the hospital system and processes. As a result of the adoption of Axiomatic Design methodology, a list of 12 Design Elements was developed. Further, the Axiomatic Design approach provides a proposal for the ideal sequence in implementing the identified Design Elements and thus helps to reduce complexity in the design of patient flows in hospitals.

This paper wants to encourage further debate, driven by studies on other possible applications of the checklist on diverse systems and on a wider scale, as well as the implementation of TRIZ to improve process reliability [29]. Moreover, it encourages additional case study-based research of the theoretical and methodological perspectives discussed so far, in order to provide new evidence of its effective applicability in diverse Health Care contexts.
